# Diagnosis and Treatment of Male Accessory Breast Cancer: A Comprehensive Systematic Review

**DOI:** 10.3389/fonc.2021.640000

**Published:** 2021-03-29

**Authors:** Liwei Pang, Meiying Cui, Wanlin Dai, Shuodong Wu, Jing Kong

**Affiliations:** ^1^ Department of General Surgery, Shengjing Hospital of China Medical University, Shenyang, China; ^2^ Department of Anesthesiology, Shengjing Hospital, China Medical University, Shenyang, China; ^3^ Innovation Institute of China Medical University, Shenyang, China

**Keywords:** accessory breast cancer, axilla, male sex, diagnosis, systematic review

## Abstract

**Background:**

Accessory breast cancer is extremely rare, especially in male patients, and only a few cases have been reported in the literature. To date, no specific guidelines regarding its diagnosis and treatment are available.

**Objectives:**

This study aimed to investigate the guidelines for the diagnosis and treatment of male accessory breast cancer by reviewing the available literature on this disease.

**Methods:**

The Web of Science, Cochrane, PubMed, and CNKI databases were systematically searched (last search: 30 November 2020) to identify studies on male axillary accessory breast cancer. The following data were extracted: author names, number of patients, country, patient age, tumor location, tumor size, pathologic diagnosis, and treatment.

**Results:**

There were 16 studies included (6 in Chinese and 10 in English), corresponding to 16 cases of male axillary accessory breast cancer. Primary surgical resection is currently the main procedure, followed by comprehensive treatment including chemotherapy, radiotherapy, and endocrine therapy. Patient age ranged from 51–87 years, and the average age was 67.1 years. The main clinical features of the patients were pain, the portion of the skin covering the mass was either reddish or purplish, and the mass could show swelling and erosion on the surface, with purulent exudate.

**Conclusions:**

Once male accessory breast cancer is diagnosed, we can follow the latest guidelines for the diagnosis and treatment of breast cancer. Tumor biopsy and resection seems the treatment of first choice, combined with comprehensive treatment including chemotherapy, radiotherapy, and endocrine therapy.

## Introduction

Accessory mammary glands, also known as aberrant mammary glands, a characteristic of polymastia, may also be associated with polythelia (1). During development, superfluous mammary glands that do not degenerate or degenerate incompletely manifest as accessory mammary glands. Accessory breasts develop from normal non-degraded breasts at an occurrence rate of 1–6% (2), and male to female ratio is approximately 1:5 (3). Similar to that in regular breasts, cancer can develop in accessory mammary glands, but it is extremely rare, especially in male patients. Accessory breast cancer is a rare form of breast cancer usually occurring in the axilla or inguinal region, where there are abundant lymph nodes and capillaries, and the incidence rate is 0.3–0.6% (4). Embryologically, mammary glands are differentiated from the same stem cells as those of the sweat glands and sebaceous glands. For this reason, it is difficult to determine whether the original site is mammary gland, sweat gland, or sebaceous gland. Owing to its rarity, there is a lack of clinical experience and specific guidelines on the diagnosis and treatment of this disease. Furthermore, early diagnosis of this disease is difficult. The 5-year survival rates of the patients with accessory breast cancer are 41.7% in the Cancer Hospital of the Chinese Academy of Medical Sciences and 35.3% in the Tianjin Cancer Hospital (5). Accessory breast carcinoma in males generally presents a worse prognosis than that in females, because the diagnosis tends to be delayed. Few reports on this disease have been published worldwide, and most of them were published in Asia. To our knowledge, there is no relevant review discussing the male accessory breast cancer systematically and comprehensively. Therefore, we performed this first systematic review on the diagnosis and treatment of male accessory breast cancer to help clinicians better understand this disease.

## Methods

This study was designed according to the preferred reporting items for systematic reviews and meta-analyses (PRISMA) guidelines. This search strategy was designed and executed by an experienced information specialist, and the retrieved articles were reviewed by two writers (MC and JK).

### Inclusion Criteria

1. Study design: clinical trials or case report.
2. Participants: Male patients diagnosed with accessory breast cancer.
3. Language: No restriction
4. Type of article: Only studies published as full text articles.

### Exclusion Criteria

1. Non-human studies, review articles, and editorials
2. Articles that did not report the outcomes of interest
3. No data on specific pathologic diagnosis

### Literature Analysis

A detailed literature search was performed using ‘accessory breast cancer’, ‘axilla’, and ‘male’ as keywords in the Web of Science (59), Cochrane (32), PubMed (69), and CNKI (80) online databases (last search date: 30 November, 2020) without regional, publication type, or language restrictions. The search strategy applied to PubMed is listed below (any keyword used in multiple forms, including its noun, adjective, or any other form, is indicated by ‘*’):

#1: accessory breast
#2: accessory mammary gland
#3: male
#4: man
#5: cancer
#6: carcinoma
#7: malignant*
#8: (#1) OR (#2)
#9: (#3) OR (#4)
#10: (#5) OR (#6) OR (#7)
#11: (#8) AND (#9) AND (#10)

When similar reports describing the same population were identified, the most recent or complete report was used. The research was conducted independently by Jing Kong and Meiying Cui, and all authors subsequently compared the results. The reference lists of articles were investigated manually, and any differences were resolved by consensus. This systematic review adhered to the guidelines outlined in the PRISMA statement.

### Data Extraction

Data on the following parameters were extracted: author names, number of patients, country, patient age, tumor location, tumor size, pathologic diagnosis, treatment, and other relevant parameters.

## Results

**Figure 1:**
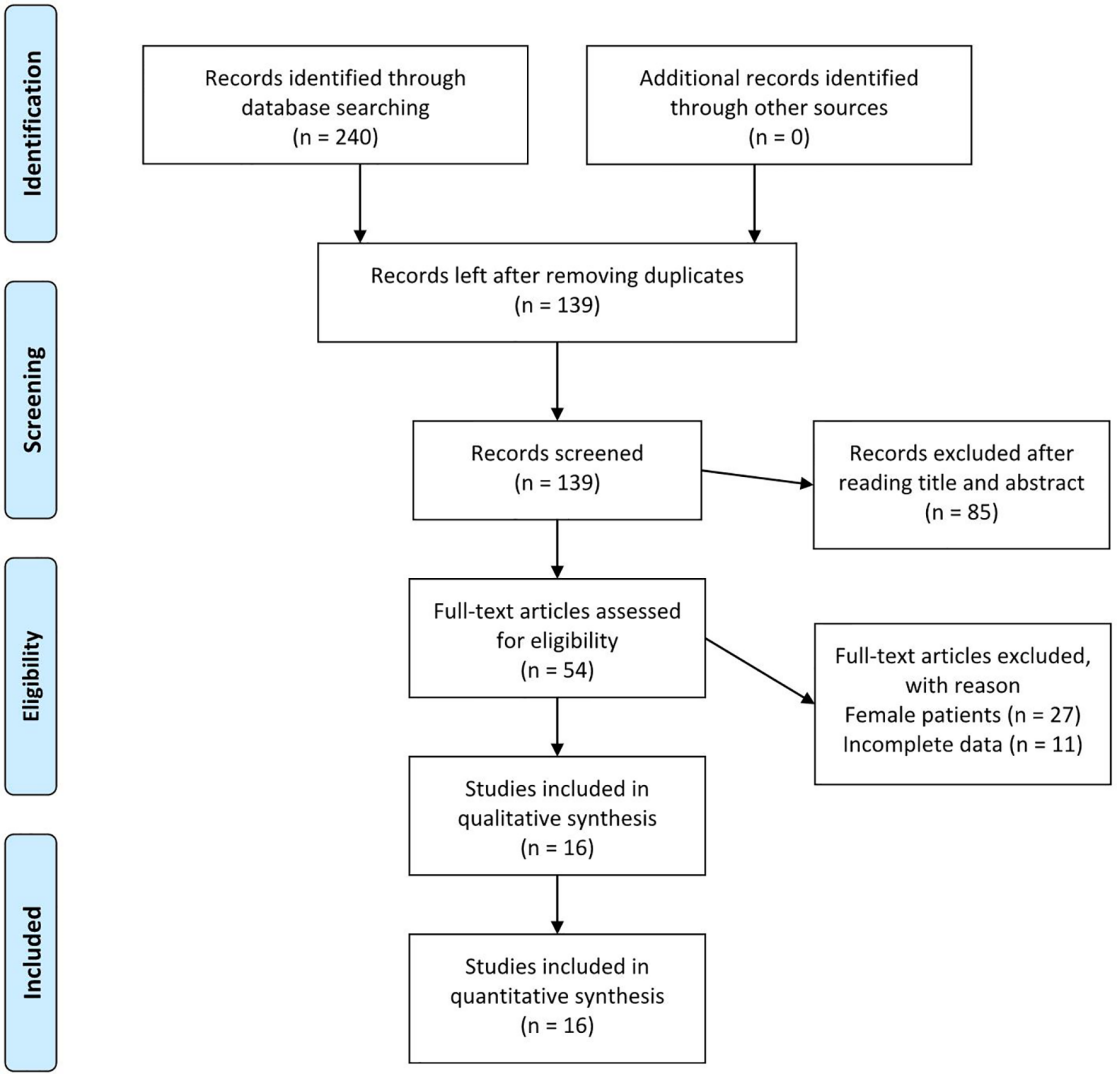
PRISMA flow chart of the literature search strategies.

Initially, 240 studies that met the search criteria were found. No other eligible studies were found from other sources. After reviewing the titles and abstracts, 54 articles were included for full-text evaluation. Eleven papers were excluded because the data were not impactful or the authors did not provide sufficient details. In addition, 27 studies were excluded because the patients described were female. Finally, 16 studies (6–21) (6 in Chinese and 10 in English, ranging from 1987–2020) corresponding to 16 cases involving male patients were included in the review. **Figure 1** illustrates the PRISMA flow chart of the literature search strategy, and **Table 1** describes the included articles.

**Table 1:** Tumor characteristics in the included articles.

Author	Year	Country	Sample size (N)	Age	Tumour location	Tumour size (mm)	Course of disease*	Clinical symptoms	Observation of malignant tumours before operation	Biopsy before operation
Gou	1987	China	1	54	Right axillary	50×50×14	2 years	Pain	Clinical suspicion	Yes
Takeyama	2010	Japan	1	58	Right axillary	80×50	5 years	Part of the skin above the mass was reddish, pain	Clinical suspicion, US, CT, MRI	No
Lin	2011	China	1	65	Right axillary	55	2 years	Part of the skin covering the mass was reddish	Clinical suspicion, US, CT, MRI, PET-CT	Yes
Yamamura	2012	Japan	1	61	Left axillary	85×51	2 years	Not mentioned	Clinical suspicion, CT, MRI, PET-CT	No
Yoshida	2012	Japan	1	73	Left axillary	80×60×70	30 years	Dark reddish protruding hard mass with an irregular surface	Clinical suspicion, CT	Yes
Xie	2013	China	1	51	Right axillary	60×40	6 months	Multiple areas of erythema	Clinical suspicion, 18F-FDG	Yes
Liu	2014	China	1	66	Right axillary	25	7 years	Not mentioned	Clinical suspicion, US, PET-CT	No
Gao	2014	China	1	68	Right axillary	35	8 years	Part of the covering skin was purplish	Clinical suspicion, US, MRI	No
Bi	2015	China	1	56	Right axillary	5	2 years	Not mentioned	Clinical suspicion, PET-CT	No
Ding	2017	China	1	69	Left axillary	40×25×20	2 months	Not mentioned	Clinical suspicion, US, CT	No
Wang	2017	China	1	87	Right axillary	12×20×17	4 years	Not mentioned	Clinical suspicion, CT, MRI, PET-CT	No
Xi	2017	China	1	87	Right axillary	23×17	5 years	Not mentioned	Clinical suspicion, US, CT, MRI	No
Liu	2018	China	1	53	Right axillary	18×7.6	8 years	Not mentioned	Clinical suspicion, US, CT, PET-CT	No
Zhong	2018	China	1	62	Right lower abdominal wall	28×25×15	More than 50 years	Pain, the mass showed swelling and erosion on the surface, with purulent exudates	Clinical suspicion, US, CT	No
Song	2019	China	1	79	Left lower abdominal wall	40×30	9 months	Part of the covering skin was purplish	Clinical suspicion, MRI	No
Bi	2020	China	1	84	Right axillary	25×24×18	More than 20 years	Pain, swelling and ulceration, bloody ulceration	Clinical suspicion, CT	No

* the time from the patient noted symptoms to treatment for the disease.

### Patient Information

Seventeen patients were identified globally, but most of them were Asian. Patient age ranged from 51–87 years, and the average age was 67.1 years. Male accessory breast cancer could develop in any possible location (3 in the left axillary region, 1 in the right lower abdominal wall, 1 in the left lower abdominal wall, and 11 in the right axillary region).

### Clinical Presentation and Examination

The clinical features of the patients were described mainly in terms of disease duration and the location and size of the tumor. In most cases, male accessory breast cancer was not associated with obvious symptoms in the early stage. With tumor growth, some patients experienced pain (4/16), and the portion of the skin covering the mass was either reddish or purplish (8/16). In the course of the disease, the mass showed swelling and erosion on the surface, with purulent exudates (2/16). The main imaging examinations included ultrasound (US), computed tomography (CT), magnetic resonance imaging (MRI), and positron emission tomography (PET)-CT. At the beginning of the disease, accessory breast cancer might show as a simple soft tissue mass or solid mass with or without enlarged lymph nodes. As the disease progressed, the cancer boundary was not clear, the echo was not uniform, the blood flow signal was punctate in the lesion, and the relationship between the mass and blood vessels was close on preoperative imaging. In addition, two (10, 13) patients were found to have organ metastasis by preoperative examination.

### Histopathology

In the included articles, the most frequent histological diagnosis was invasive ductal carcinoma (7/16), followed by poorly differentiated adenocarcinoma (5/16). The results of immunohistochemistry for the estrogen receptor (ER), progesterone receptor (PR), and human epidermal growth factor receptor 2 (HER2) are shown in **Table 2**. Among 15 patients with histological diagnoses, 10 were ER-positive (66.7%), 9 were PR-positive (60%), and 5 were positive for HER2 (33.3%).

**Table 2:** Pathology, treatment, and patient prognosis in the included literature.

Author	Year	Organ metastasis (before operation)	Pathologic diagnosis	Immuno-histochemistry*	Lymph node invasion (postoperative pathology)	Treatment	TNM stage**	Outcome
Gou	1987	Not mentioned	Intraductal carcinoma	Not mentioned	Not mentioned	Lymphadenectomy	Not mentioned	Followed-up for 3 years without recurrence
Takeyama	2010	Not mentioned	Poorly differentiated adenocarcinoma metastatic from an unknown primary	Lactalbumin(-), CA19-9(-), CA125(-), CEA(-), PAS(-), Al Blue(-), ER(+), PR(+)	Yes	Chemotherapy, and tumour resection with axillary lymph node dissection	T3N1M0	Not mentioned
Lin	2012	Not mentioned	Moderately differentiated adenocarcinoma	CK7(-), CK20(-), S-100(-), p63(-), HER2(-), EMA(-), GCDFP15(+), Ki-67(+), ER(+), PR(+)	Yes (4/21)	Incisional biopsy of the mass, adjuvant radiotherapy between the four cycles of AC and four cycles of paclitaxel	T3N2M0	Free from recurrence on his last follow-up visit 18 months after surgery
Yamamura	2012	Not mentioned	Adenocarcinoma compatible with breast carcinoma originating in an accessory mammary gland	Ki-67(-), ER(+), PR(+), HER2(-)	Not mentioned	Neoadjuvant chemotherapy, complete resection	T3N0M0	No metastatic lesion in 4 years
Yoshida	2012	Cerebellar and bone metastases	Marked proliferation of atypical tumour cells forming nests with adenoid structures	ER(-), PR(-), HER2(+)	Yes (24/39)	Skin biopsy, trastuzumab	T3N2M1	Brain metastases developed, and the patient died 6 months after the operation
Xie	2013	Not mentioned	Poorly differentiated adenocarcinoma	CK7(+), CKpan(+), GCDFP15(-), ER(-), PR(-), S-100(-)	Yes	Lesion biopsy, chemotherapy	T3N1M0	Not mentioned
Liu	2014	Thoracic metastasis	Invasive ductal carcinoma	ER(-), PR(+), HER2(-)	Yes	Radical mastectomy	T2N1M0	Not mentioned
Gao	2014	No	Moderately differentiated adenocarcinoma	ER(+), PR(+), HER2(+)	Yes	Tumour resection with axillary lymph node dissection	T2N1M0	Not mentioned
Bi	2015	No	Poorly differentiated adenocarcinoma	CK20(-), ER(+++), HER2(-), TTF-1(-), GCDFP15(+)	Yes (3/17)	Right breast modified radical mastectomy and left breast simple excision, six cycles of adjuvant chemotherapy	T2N2M0	Followed-up for 4 years with a recurrence
Ding	2017	No	Invasive ductal adenocarcinoma	ER(-), PR(-), EMR(-), HER2(-), p63(-), CK7(+), CK8(+), CD117(-), E-Cad(+)	No (0/17)	Extended resection of left axillary lesions and axillary lymph node dissection, chemotherapy	T2N0M0	Not mentioned
Wang	2017	No	Right axillary mucinous adenocarcinoma	ER(+), PR(+), HER2(+), p120(+), CK7(+), CK20(-), villin(-), CEA(-), TTF-1(-), napsin A(-)	No (0/3)	Right accessory breast cancer radical surgery, endocrine therapy	T1N0M0	Followed-up for 2 years without recurrence
Xi	2017	No	Mucinous adenocarcinoma	ER(+), PR(+), HER2(-), Ki-67(+)	No (0/3)	Extended resection of right axillary lesions and axillary lymph node dissection, chemotherapy	T2N0M0	Followed-up for 2 years without recurrence
Liu	2018	Not mentioned	Invasive lobular carcinoma	ER(-), PR(-), HER2(-), CK(+), CA153(+), p120(+), E-Cad(+), vimentin(+), Ki-67(+)	Yes (16/18)	Extended resection of right axillary lesions, chemotherapy	T2N2M0	Followed up for 1 year without recurrence
Zhong	2018	Not mentioned	Grade II infiltrating ductal carcinoma	ER(+), PR(+), HER2(-), Ki-67 (40% positive), Syn(+), CgA(+), GCDFP15(+)	Yes	Abdominal mass resection	T2N1M0	Not mentioned
Song	2019	Not mentioned	Invasive ductal carcinoma	ER(+), PR(-), HER2(++)	Yes	Biopsy of inguinal lymph nodes	T2N1M0	Not mentioned
Bi	2020	Not mentioned	Grade II infiltrating ductal carcinoma	ER(++), PR(+++), HER2(1+), Ki-67(+)	Yes	Modified radical mastectomy and Axillary lymph nodes clearance, endocrine therapy	T2N1M0	No signs of recurrence

* (-), negative; (+), positive; CEA, carcinoembryonic antigen; PAS, periodic acid Schiff reaction; ER, estrogen receptor; EMA, epithelial membrane antigen; GCDFP15, gross cystic disease fluid protein 15; E-Cad, E-Cadherin; HER2, Human epidermal growth factor receptor-2. ** the clinical stage (TNM) of each accessory breast cancer according to the breast cancer.

### Lymph Node Invasion and Organ Metastasis

Similar to that in breast cancer, homolateral axillary nodes were the most common sites of lymphatic metastasis. In addition, accessory breast cancer showed involvement of the ipsilateral supraclavicular nodes. Among the 16 patients, 11 were confirmed to show metastasis to at least one lymph node. Furthermore, accessory breast cancer could have characteristics of malignant tumors such as neighbor organ invasion and metastasis, including thoracic metastasis (one case) and cerebellar and bone metastases (one case). These two patients had a longer disease course (7 and 50 years). Organ metastasis from accessory breast cancer was rare unless the long disease course.

### Treatment and Prognosis

Currently, no specific guidelines on the diagnosis and treatment of male accessory breast cancer are available. In the included axillary cases, the main treatment used included simple tumor resection (5 cases), tumor resection with axillary lymph node dissection (5 cases), radical mastectomy (3 cases), chemotherapy (7 cases), and endocrine therapy (2 cases). In the abdominal wall cases, the patients were treated by abdominal mass resection (1 case) or biopsy and resection of inguinal lymph nodes (1 case). In the last century, the main treatment for male accessory breast cancer was simple tumor resection and lymph node dissection. With developing understanding of the underlying disease, the treatment of first choice seems to be tumor biopsy and resection combined with comprehensive treatment including chemotherapy, radiotherapy, and endocrine therapy. Furthermore, there is no consensus on whether prophylactic excision of the ipsilateral breast is necessary to prevent this malignancy. Among the 16 patients, 3 returned to the hospital due to tumor recurrence or metastasis. However, due to inconsistency in data regarding operation and follow-up, there is no specific evidence to show the relationship between the surgical procedure and prognosis. Nevertheless, we were able to identify that these 3 patients had definite early lymph node metastasis (**Table 2**).

## Discussion

The predisposing occurring site of accessory breast cancer is axilla, followed by groin. Male accessory breasts may appear at any site along the milk line from the axilla to the groin due to incomplete embryologic regression of the mammary ridge (22). The first case of male accessory breast cancer was reported in 1957 in Russia (23), but few subsequent cases have been reported. In recent years, with the development of pathology and imaging technology, the number of cases of male accessory breast cancer identified and reported has increased. In this systematic review, we searched all the relevant literature around the world and concluded the basic information, symptoms, treatment, and prognosis of all patients to help clinicians better understand this disease.

In the early stages of male accessory breast cancer, patients either show no symptoms or only harbor a small mass. With growth, accessory breast cancer tumors could compress and invade nerves, which causes pain and affects physical activity. If the skin is invaded by the tumor, it becomes reddish or purplish in color, the region becomes swollen, and an ‘orange peel’ appearance is observed (24). This stage occurs a long time after disease onset. Unfortunately, patients commonly choose to visit a doctor only at this stage, resulting in delayed diagnosis.

The diagnosis of accessory breast cancer is challenging. In the early stage of the disease, accessory breast cancer only presents as a painless mass. As the disease progresses, the main clinical symptoms of this disease include pain, reddish or purplish skin, swelling, and an eroding mass. This is also the most common time for patients to visit a doctor. For the attending physician, it is first necessary to determine whether an observed mass is a primary lesion or a metastatic epithelial tumor. Primary lesions generally include sebaceous gland or sweat gland cancer and accessory breast cancer; metastatic lesions usually originate from the breast, gastrointestinal tract, prostate, lung, and some metastatic lesions have lymph node metastasis (25, 26). The axillary region is a well-developed apocrine gland. Consequently, the clinical manifestations of accessory breast cancer and sweat gland cancer are also similar. Apocrine gland carcinoma should be considered in the differential diagnosis if malignant tumors are observed in this region. Using imaging examinations, such as US, CT, MRI, or PET-CT, primary malignant tumors in other organs can be excluded. Among the related auxiliary examinations, US may be preferred (19, 27). The US findings of accessory breast cancer typically include a hypoechoic mass with uneven echogenicity and a crab foot-shaped edge (27). The value of CT in diagnosis is not yet clear, but this method is useful for differential diagnosis, for staging assessment (9, 19), and for ruling out metastatic carcinoma. On MRI scans, accessory breast cancer has good soft tissue resolution and high-contrast resolution, which clearly show the location and extent of lesions and the relationship with the surrounding tissues and organs (19, 28, 29). ^18^F-FDG PET-CT is performed to localize the primary lesion and for systemic evaluation (30). Different physical examinations have different advantages. In short, the absence of metastatic carcinoma, lack of continuity with the normal breast tissue, existence of normal breast tissue around the carcinoma, and the absence of sudoriparous carcinoma in the axilla are the important criteria for diagnosis (30). Based on these criteria, a biopsy or tumor excision should be performed, and pathological examination remains the gold standard for the diagnosis of accessory breast cancer. If the diagnosis had been delayed, the tumors might have grown further and resulted in a more-advanced tumor stage and a higher lymph node stage. This would require more-extensive surgery, and potentially lead to worse outcomes, or result in morbidity, due to systemic therapy or limited survival.

The most frequent histological type of this lesion is invasive ductal carcinoma. The homolateral axillary nodes were the most common sites of lymphatic metastasis. However, in the included patients, no patient underwent sentinel lymph node biopsy. The main treatment of axillary lymph nodes is resection or biopsy. For the two abdominal wall cases (19, 20), one patient underwent abdominal lymph node resection (19) and the other patient (20) underwent biopsy and resection of inguinal lymph nodes. In addition to the typical histological characteristics of breast cancer, the expression of breast-derived immunohistochemical markers can also be used to further confirm the diagnosis (19), although male accessory breast cancer itself does not have special immunohistochemical markers. ER, PR, HER2, and other makers are often used only to guide treatment and predict prognosis. Yamamura et al. (9) reported a case of male breast cancer originating in an accessory mammary gland in the axilla, which was successfully treated using hormone therapy (tamoxifen at 20 mg/day). The tumor had the following immunohistochemical characteristics: Ki-67(-), ER(+), PR(+), and HER2(-). In addition, Wand et al. (16) reported a case of accessory male breast cancer in which an 87-year-old male patient was treated with hormone therapy. Considering the chemotherapy-related toxicity, adjuvant chemotherapy was not considered suitable for this patient (tamoxifen at 20 mg/day). This tumor showed the following characteristics: ER(+) >75%, PR(+) >75%, HER2(++), p120(+), cytokeratin 7(+), cytokeratin 20(-), villin(-), carcinoembryonic antigen(-), thyroid transcription factor-1 (TTF-1)(-), and napsin A(-). Bi et al. (14) reported a case of locally advanced male accessory breast cancer with delayed diagnosis that was successfully treated with endocrine therapy (docetaxel, 140 mg intravenously and cyclophosphamide, 1 g intravenously on day 1 then once every 3 week). The findings in this patient were: CK20(-), ER(+++), HER2(-), TTF-1(-), and gross cystic disease fluid protein 15(focal+). Nevertheless, in 2 cases (11, 14), recurrence or metastasis occurred after treatment; one of these tumors was ER(-), PR(-), HER2(+) and the other was ER(-), PR(+), HER2(-) (**Table 2**).

Lymph node status is an important prognostic factor in breast cancer and might be in male accessory breast cancer as well. The anatomy of lymphatic drainage of the accessory breast is unclear. Therefore, the role of sentinel lymph node biopsy is important. The sentinel node is the first lymph node to receive lymphatic drainage from a tumor; and, as such, it will be the first site of metastasis if lymphatic dissemination has occurred. Sentinel lymph node biopsy allows for more-accurate staging of the disease; and, in doing so, allows more-appropriate adjuvant treatment. Thorne et al. (31) thought the use of lymphatic mapping and sentinel node biopsy in the case of a female accessory breast cancer allowed more-accurate determination of the lymph node status. Even with the history of excision of the primary lesion, sentinel node mapping is still a viable method for lymph node staging in ectopic axillary breast cancers (32).

Because of the rarity of accessory breast cancer, the diagnostic procedure and treatment strategy for accessory breast cancer have not been established (33). The treatment of male accessory breast cancer is similar to that of regular breast cancer and follows the principle of comprehensive treatment, with surgery as the main treatment, supplemented with chemotherapy, radiotherapy, endocrine therapy, and molecular targeted therapy. Operations include radical resection, extended lesion resection (local extended lumpectomy and flap transplantation), lymph node dissection, and ipsilateral breast modified radical mastectomy. Madej et al. (34) suggested that if the accessory mammary gland is anatomically located very close to the normal breast, clinicians should perform modified radical mastectomy, and if the accessory breast cancer is located far away from the breast, resection of the normal breast is not necessary and only radical accessory breast resection is sufficient. After Evans (35) reviewed 90 cases of carcinoma of ectopic breast tissue, no survival advantage for radical or modified radical mastectomy over local excision combined with axillary dissection or radiation was found.

**Figure 2:**
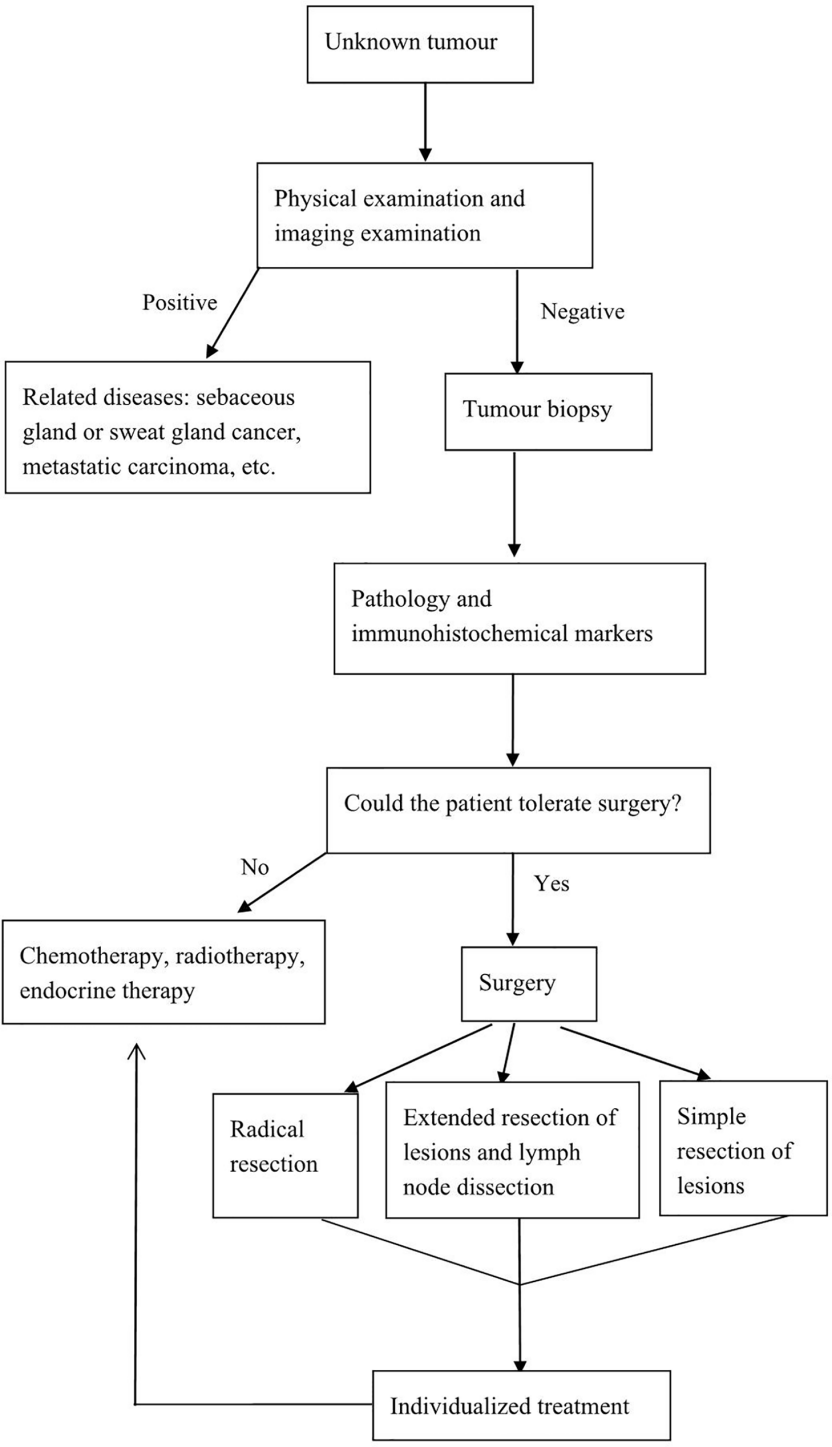
Flow diagram of the diagnosis and treatment process.

Postoperative adjuvant therapy has not been standardized. Chemotherapy, radiotherapy, endocrine therapy, or targeted drug therapy should be selected wisely based on disease stage, immunohistochemical examination results, and the metastatic status of lymph nodes (36). In patients who cannot tolerate surgery or who refuse surgery, radiotherapy can provide good local control in case of limited tumors combined with only minimal side effects and chemotherapy or endocrine therapy can eliminate the tumor or reduce the tumor size in advanced disease (10, 15, 17). To facilitate the management of this disease, we have developed a relatively feasible diagnostic and treatment process (**Figure 2**).

Because the occurrence of male accessory breast cancer is extremely rare, the prognosis of accessory breast carcinoma is difficult to establish, primarily because of absent or limited follow-up data. Among the 16 patients enrolled in this study, 3 were definitively diagnosed as having recurrence or metastasis. Japanese scholars (37) followed up 68 cases, with a mean follow-up duration of 28.3 months (range, 2–156 months), and only one patient died due to pneumonia during the perioperative period. Youn (38) also found that the prognosis of accessory breast cancer was not worse than that of breast cancer in the same time period. Therefore, the prognosis in patients with accessory breast cancer is relatively good (39, 40).

## Limitations

The advantage of this review is that it provides a comprehensive review of male accessory breast cancer. To our knowledge, this is the first systematic review to explore the diagnosis and treatment of male accessory breast cancer. Of course, this review has some limitations, which should be noted. First, publication and selection bias could be a substantial issue because of most of the patients came from Asia. Second, the small number of patients and studies decreased the reliability, even though we searched through several databases. Furthermore, the size of this study was not large enough, all included studies were case reports, and the results need more-effective evidence from further high-quality trials.

## Conclusion

Although accessory breast cancer in males is extremely rare, the possibility of this disease should be considered when establishing a diagnosis. Once male accessory breast cancer has been diagnosed, the latest guidelines for the diagnosis and treatment of breast cancer can be followed. Currently, primary surgical resection supplemented with comprehensive treatment including chemotherapy, radiotherapy, and endocrine therapy is the main treatment for male accessory breast cancer. Moreover, we hope the public have a deeper understanding of the disease through this review.

## Data Availability Statement

The original contributions presented in the study are included in the article/supplementary material. Further inquiries can be directed to the corresponding author.

## Author Contributions

LP: literature research, manuscript preparation; MC: literature research, manuscript preparation; WD: literature research; SW: literature research; JK manuscript final version approval. All authors contributed to the article and approved the submitted version.

## Funding

This study was funded by the National Nature Science of China (81670580) and 345 Talent Project and Shenyang Science and Technology Innovation Talent Support Program for Youth and Midlife (RC200121).

## Conflict of Interest

The authors declare that the research was conducted in the absence of any commercial or financial relationships that could be construed as a potential conflict of interest.
